# A refined experimental model for local, interpolated flap, and free tissue transfer studies using musculus cutaneus maximus-based musculocutaneous flap in the rat

**DOI:** 10.1590/acb408125

**Published:** 2025-11-10

**Authors:** Gergo Kincses, Laszlo Adam Fazekas, Adam Varga, Adam Attila Matrai, Anna Orsolya Flasko, David Martin Adorjan, Abel Molnar, Adam Deak, Norbert Nemeth

**Affiliations:** 1 University of Debrecen – Faculty of Medicine – Department of Surgery – Debrecen – Hungary.; 2 University of Debrecen – Faculty of Medicine – Department of Operative Techniques and Surgical Research – Debrecen – Hungary.; 3 University of Debrecen – Faculty of Medicine – Department of Otorhinolaryngology and Head and Neck Surgery – Debrecen – Hungary.; 4 University of Debrecen – Faculty of Medicine – Department of Dermatology – Debrecen – Hungary.

**Keywords:** Myocutaneous Flap, Models, Theoretical, Perfusion, Microcirculation

## Abstract

**Purpose:**

Adequate microcirculation is essential for regeneration and survival of flaps. The perfusion pattern can be influenced by the vascular pedicle’s properties in various flap types. We aimed to describe a refined musculocutaneous flap model with viability measurements, studying local, interpolated and transferred (free) flap types in rats.

**Methods:**

Wistar rats were subjected to three experimental groups (n = 8/each). Beside controls, in two groups cutaneous maximus musculocutaneous flaps were prepared bilaterally. The right flaps were sutured back (local flap), the left one was transposed to the frontal chest wall (interpolated flap). In another group, left flap was transferred to the inguinal region performing microvascular anastomoses. Flaps’ temperature, blood flow, and microcirculation were assessed before/after operation and on the 14^th^ postoperative day.

**Results:**

The flaps’ temperature didn’t worsen, but values moderately decreased in transferred flaps. The pedicle’s blood flow didn’t change significantly after preparation. The transferred flaps’ values lowered by the 14^th^ day. Microcirculatory parameters decreased postoperatively, significantly in interpolated and transferred flaps, and completely normalized by the 14^th^ day.

**Conclusion:**

Tissue perfusion and microcirculatory pattern were sufficient for flap survival and wound healing. The refined cutaneous maximus musculocutaneous flap model can be useful in studies comparing local, interpolated and transferred flaps.

## Introduction

In many reconstructive surgical procedures, flaps of various compositions, shapes, sizes, and types play an important role in covering tissue defects originating from injuries, ulcerations, necrosis, or radical oncological surgeries^1^^–^ 3. Concerning the complications and conditions leading to flap failure, experimental surgical research is still important^4^. For studying flap pathophysiology, optimizing flap design, preconditioning, and preventive possibilities, numerous experimental models of various flaps (location, tissue composition) have been published^5^^,^ 6. Depending on the investigative protocol and instrumentation, small and large animal models may provide skin-, fasciocutaneous-, musculocutaneous-, muscle-, and other composite flap designs^5^^–^ 17. To choose the appropriate model, it is important to link the main scientific question to the clinical relevance, but also to the laboratory animal science issues, animal welfare, available investigative methods, operative skills, as well as the duration and circumstances of the follow-up period.

Although the main complications of flap surgery, such as necrosis, perfusion, and microcirculatory disturbances, ischemia/hypoperfusion, venous congestion, suture insufficiency, and infection are well recognized, numerous questions remain unanswered^18^^–^ 22. The exact perfusion thresholds and microcirculatory changes that predict flap survival must be fully clarified yet, and there is a lack of reliable early indicators of ischemia or venous congestion. The tolerance of different flap types to ischemia and the molecular mechanisms underlying ischemia–reperfusion injury require further investigation, as does distinguishing between arterial and venous compromise in clinical settings. Technical aspects, including the biomechanical properties of suture materials and anastomotic stability, continue to be a cause for concern, while the interplay between bacterial contamination, immune responses, and impaired wound healing is not fully understood. Furthermore, the impact of flap design and geometry, particularly the pedicle-to-flap size ratio, orientation, and thickness, on perfusion gradients and necrosis development, requires systematic evaluation^1^^–^ 3 ^,^ 18 ^,^ 19 ^,^ 23.

In previous studies, we have investigated microcirculatory, micro-rheological, and histomorphological features of various flaps in small and large animal models related to ischemia–reperfusion injury^8^^,^ 9 ^,^ 11 ^,^ 14 ^,^ 15. Based on these experiences, we wished to use a flap model that can be standardized well and that is appropriate to be used for studying local, transpositioned (interpolated), and transferred (free flap). In this paper, we aimed to describe a refined musculocutaneous flap model based on the cutaneous maximus muscle in rats with related flap viability measurements.

## Methods

### Experimental animals and groups

The study was conducted under national regulation (Act XXVIII of 1998 on the Protection and Humane Treatment of Animals) and European Union (EU) Directive (2010/63/EU). The experiment has been registered and approved by the University of Debrecen Committee of Animal Welfare (registration number: 19/2022/UDCAW) and the National Food Chain Safety Office.

Twenty-four male Wistar rats (Crl:WI, Toxi-Coop Zrt., Budapest, Hungary; bodyweight: 351.3 ± 49.1 g) were involved in the study. The animals were housed in the department’s animal facility (conventional status, cages: Eurostandard IV, Tecniplast, Buguggiate, Italy; temperature 22 ± 2°C; humidity 55 ± 10%; lighting 12–12-h light/dark cycle), had free access to water and standard rodent chow (SAFE^®^ D132, Complete Care Competence, Augy, France). After the operation, the animals were housed individually.

Using computer-generated random numbers of individual identification codes, the animals were randomized into three experimental groups: sham-operated control (control group, n = 8), group with local and interpolated flaps (Flap group, n = 8), and group with local and transferred flaps (TransFlap group, n = 8).

In the control group, anesthesia, skin depilation bilaterally above the relevant territories, and immobilization for the operation time (similarly to other groups) were the elements of the protocol.

In the flap group, musculocutaneous flaps based on the musculus cutaneous maximus were prepared bilaterally. One flap was sutured back to its original position (right), while the other one was interpolated to the front wall of the chest through a subcutaneous tunnel (left).

**Figure 1:**
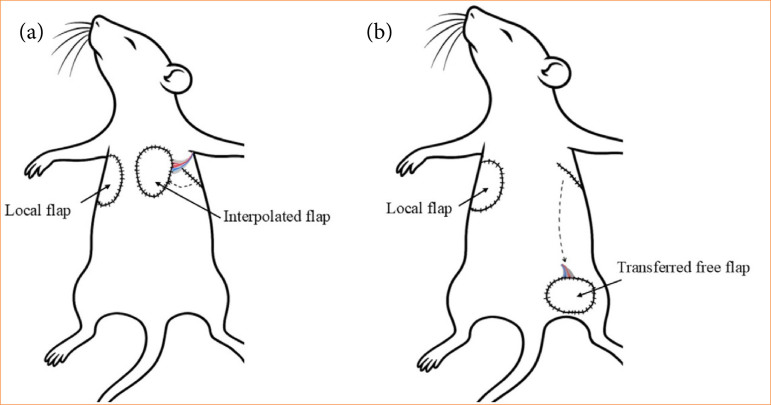
Schematic drawing of the experimental design in the group with local and interpolated flaps (flap group), and the group with local and transferred flaps (TransFlap group).

Source: Elaborated by the authors.

In the TransFlap group, besides forming a local one, the other flap was transferred to the left inguinal region (as a free flap), performing microvascular anastomoses (Fig. 1; Table 1).

**Table 1:** Overview of experimental groups and flap types.

Group	Side	Flap type
Control	Right	None
	Left	None
Flap	Right	Local
	Left	Interpolated
TransFlap	Right	Local
	Left	Transferred

Source: Elaborated by the authors.

### Operative techniques in detail

General anesthesia was provided using ketamine hydrochloride (100 mg/bwkg, intraperitoneally, CP ketamine hydrochloride 10%, Produlab Pharma BV, Raamsdonksveer, The Netherlands) and xylazine hydrochloride (10 mg/bwkg, intraperitoneally, CP xylazine hydrochloride, 2%; Produlab Pharma BV, Raamsdonksveer, The Netherlands)^24^^,^ 25. During anesthesia, respiratory rate and depth (spontaneous ventilation), color of the mucous membrane and extremities were observed, and a heating pad was used to maintain body temperature. Animals were laid in a supine anterolateral position.

For harvesting the musculocutaneous flap, the M. cutaneous maximus (supplied by the lateral thoracic artery) and the covering fascia layer and skin were used bilaterally. After depilation and before flap preparation, the angiosome extension was estimated using a CytoCam-IDF camera (Braedius Medical, Huizen, the Netherlands). A circular plastic sheet (surface area = 7.13 cm^2^) was used as a standard bean-shaped template to sign the contour of the flap for proper skin incision and preparation. The convexity looked toward the back, and the cranial apex was positioned in the frontal part of the armpit. The skin in the thoracic region is relatively thin, so the incision had to be accurate and carefully performed.

The cutaneous maximus muscle is a skin muscle: the skin gets some part of its blood supply from the muscle. Therefore, completely dissecting the muscle from the skin was avoided to preserve the blood supply. We left a 5–6-mm wide muscle edge around the flap. The remaining muscle tissue’s vitality was satisfying due to the collateral circulation. Therefore, finding an adequate preparation layer was important. Under the muscle layer, the supplying vessels were covered by a fatty pad. To avoid vessel damage, we separated this pad and left it on the thoracic wall. The supplying vessels and the axillary region are also surrounded by fatty tissues. The strongest blood flow is under the side branches, in which we performed the blood flow measurements. The harvested flaps were covered with wet gauze (body-temperature physiological saline solution).

In case of local flaps (right), we sutured them back to their original anatomical position with tension-free, anchoring simple interrupted stitches, and intracutaneous sutures between those stitches using 4-0 polypropylene suture material with reverse cutting needle (Pidelen; KOLLSUT, Hauppauge, NY, United States of America).

When preparing the interpolated flaps, an iatrogenic skin and soft tissue defect was created on the left frontal chest wall. Under the skin bridge, we formed a tunnel that was wide enough for a tension-free flap interpolation. Then, we gently pulled the flap through the recipient site. During the process, torsion, twisting, and overstretching of the flap vascular pedicle had to be avoided. The subcutaneous tunnel also served as a protective layer from external damage. The suturing was like the local flaps. Additionally, we also performed the primary closure of the donor site.

**Figure 2:**
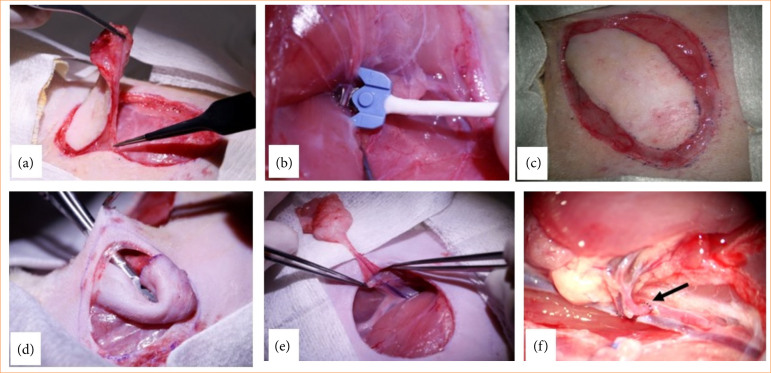
Main steps of the experimental operation: preparation of the musculus cutaneus maximus: **(a)** based musculocutaneous flap, **(b)** measurement of blood flow on the artery (lateral thoracic artery branch) of the flap pedicle, **(c)** the prepared flap left locally, **(d)** transposition of the flap (interpolated) via a subcutaneous tunnel, **(e)** positioning the free flap in the femoro-inguinal region, and **(f)** microvascular anastomosis between the flap pedicle vessels and the femoral artery and vein.

Source: Elaborated by the authors.

The femoro-inguinal region was appropriate to receive the free (transferred) flap. The relatively long flap pedicle makes it ideal for free tissue transfer. The diameter of the supplying vessels is under 1 mm (artery = 0.53 ± 0.06; vein = 0.91 ± 0.11). Therefore, microsurgical techniques were needed to perform vascular anastomoses. An iatrogenic skin and soft tissue defect was made in the inguinal region, in which the free flap was transferred. The superficial epigastric artery and vein were gently prepared until their origin from the femoral vessels. The femoral artery was clipped after the Murphy’s branch and after the epigastric artery. Because of the discrepancy between the flap pedicles’ vessels and the epigastric vessels, we elevated the femoral vessels’ wall with the branches and then cut out a hole with a proper diameter for the end-to-side anastomosis. After forming the anastomosis orifices and flushing the lumen with diluted heparin sodium solution, end-to-side anastomoses were performed (first the artery)^26^^,^ 27 between the flap pedicle vessels and the femoral artery. While the flaps were transferred, we perfused the flaps with heparin solution via the pedicle vessels. The vascular anastomoses were performed using interrupted microsutures with 11-0 monofil non-absorbable poliamide-6 suture material and serosa (taper) needle (Daclon, SMI, Vith, Belgium). We kept the anastomosis suturing time under 20 minutes. Patency was proven by the milking test^28^. In the case of the vein, we used similar techniques. Skin closure happened similarly to the other flap types. The main steps of the operations are shown in Fig. 2.

Animals had a plastic collar around their neck to prevent autophagy, daily wound control was carried out for two weeks, and Tramadol (15 mg/bwkg/day) was given daily on the first to the third postoperative days^29^. The follow-up period was 14 days. At the end of the experiment, animals were anesthetized, measurements were completed, and they were euthanized, giving an overdose of anesthetics (50 mg/bwkg ketamine and 5 mg/bwkg xylazine) through the tail vein cannula.

### Flap viability investigations

We measured skin surface temperature (rodent NIBP infrared thermometer with LaserSight, ADInstruments, United States of America) on all the flaps and on the intact abdominal skin region (above the xiphoid process of the sternum) before flap preparation, just after their resuturing, as well as on the 14^th^ postoperative day.

The blood flow (mL/min) in the flap pedicle was tested using a Transonic T206 device (Transonic Microcirculation Flowprobe; Transonic Systems Inc., Ithaca, NY, United States of America)^30^. The ultrasound probe of the device was gently placed around the supplying pedicle’s artery. We performed the measurements after flap harvesting and just before suturing the wounds, as well as on the 14th postoperative day.

Skin microcirculation was monitored with a CytoCam-IDF camera (Braedius Medical, Huizen, the Netherlands). Recordings were made before (base) and just after the operation, and on the 14^th^ postoperative day. The method is based on incident dark field illumination technology, providing real-time visualization of the microcirculation^31^^,^ 32. With the help of the device’s software (CytoCamTools V3 Bedside Manager, Braedius Medical, Huizen, the Netherlands), we analyzed the high-resolution video recordings off-line, determining the microvascular flow index (MFI [au]), the proportion of perfused vessels (PPV [%]), the perfused vessel density (PVD [mm/mm^2^], and general parameters^31^^–^ 33.

### Statistical analyses

Statistical analyses were completed using SigmaStat Software 3.1.1.0 (Systat Software Inc., San Jose, CA, United States of America). Data are shown as means ± standard deviation (SD). For inter-group comparisons, t-test or Mann-Whitney rank sum test were used, and for intra-group comparisons one-way analysis of variance (ANOVA) or Kruskal-Wallis tests, depending on the normality of data distribution. *P* < 0.05 was considered statistically significant.

## Results

All the flaps survived well, and we did not experience any serious complications leading to flap failure. No swelling, necrosis, or infection was observed.

Skin temperature of the local and interpolated flaps did not alter significantly right after the operation and by the 14^th^ postoperative day. In case of transferred flap, skin temperature decreased with an average of 4.77% right after operation and was still lower (3.47% *versus* base) at the end of the follow-up period (Table 2).

**Table 2:** Skin temperature of intact abdominal skin region, right (local) and left (interpolated or transferred, according to the groups) flaps, tested before (base) and just after the operation and on the 14^th^ postoperative (p.o.) day*.

Region	Group	Base (°C)	Just after operation (°C)	14^th^ p.o. day (°C)
Abdomen	Control	36.13 ± 0.49	36.02 ± 0.52	35.75 ± 0.50
	Flap	36.27 ± 0.33	35.41 ± 0.92	36.02 ± 0.57
	TransFlap	36.33 ± 0.44	36.43 ± 0.13	36.15 ± 0.53
Local (right) flap	Control	35.84 ± 0.62	35.76 ± 0.61	35.13 ± 0.88
	Flap	35.78 ± 0.55	35.41 ± 0.63	35.95 ± 0.57
	TransFlap	36.15 ± 0.53	35.63 ± 0.68	35.33 ± 0.57
Interpolated (left) flap	Flap	35.76 ± 0.55	35.20 ± 0.82	35.84 ± 0.72
Transferred (left) flap	TransFlap	36.26 ± 0.51	34.53 ± 0.32	35.00 ± 0.83**

* means ± standard deviation;

** *p* < 0.05 *versus* base.

Source: Elaborated by the authors.

The arterial blood flow (mL/min) of the flap pedicle did not differ significantly after preparing the flaps. However, the highest blood flow rate values were seen in the case of local flaps, in which the original anatomical position was maintained. We observed that the arterial blood flow rate values were significantly lower on the 14th postoperative day in all the flap types. The lowest values were seen in the transferred flaps (*p* = 0.039) (Table 3).

**Table 3:** Flap pedicle’s arterial blood flow rate measured after harvesting the flaps and on the 14^th^ postoperative (p.o.) day in experimental groups with various flap types*.

Flap	Group	Base [mL/min]	14^th^ p.o. day [mL/min]	*p*-value
Local (right) flap	Flap	0.60 ± 0.17	0.40 ± 0.15**	0.015
	TransFlap	0.46 ± 0.12	0.43 ± 0.13	n.s.
Interpolated (left) flap	Flap	0.61 ± 0.16	0.37 ± 0.12**	< 0.001
Transferred (left) flap	TransFlap	0.49 ± 0.11	0.35 ± 0.13**	0.039

* Means ± standard deviation;

** *p* < 0.05 *versus* base.

Source: Elaborated by the authors.

**Figure 3:**
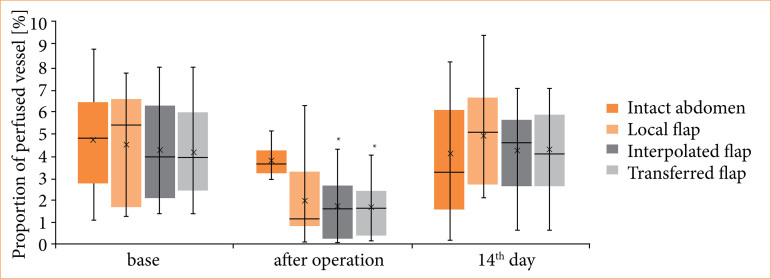
Microcirculatory proportion of perfused vessel (%) parameter of intact abdominal skin region, local, interpolated, and transferred flaps, tested before (base) and just after the operation, and on the 14th postoperative day*.

The microvascular flow index values were above 2 in all cases (2.72–2.84), so the video recordings could be analyzed well to safely assess further parameters. The PVD was unchanged in the control skin region in all groups. In local, interpolated, and transferred flaps, PVD values non-significantly decreased just after the operation, and the values were lower on the 14^th^ postoperative day as well. However, the differences were not significant (Table 4). The values of PPV decreased just after the operation, significantly in the interpolated (*p* = 0.001 versus base) and transferred flap (*p* < 0.001 versus base). The values by the 14th postoperative day were like the base ones (Fig. 3).

*p < 0.05 versus base. Source: Elaborated by the authors.

**Table 4:** Microcirculatory perfused vessel density (mm/mm2) parameter of intact abdominal skin region, right (local), and left (interpolated or transferred, according to the groups) flaps, tested before (base) and just after the operation and on the 14^th^ postoperative (p.o.) day*.

Region	Group	Base [mm/mm2]	Just after operation [mm/mm^2^]	14^th^ p.o. day [mm/mm^2^]
Abdomen	Control	0.77 ± 0.49	0.81 ± 0.74	0.81 ± 0.74
	Flap	0.75 ± 0.57	0.78 ± 0.64	0.79 ± 0.66
	TransFlap	0.76 ± 0.43	0.79 ± 0.61	0.79 ± 0.62
Local (right) flap	Control	0.78 ± 0.97	0.49 ± 0.44	0.46 ± 0.35
	Flap	0.74 ± 0.93	0.49 ± 0.45	0.45 ± 0.36
	TransFlap	0.64 ± 0.89	0.50 ± 0.45	0.45 ± 0.35
Interpolated (left) flap	Flap	0.81 ± 0.50	0.42 ± 0.47	0.41 ± 0.47
Transferred (left) flap	TransFlap	0.62 ± 0.68	0.41 ± 0.36	0.42 ± 0.36

* Means ± standard deviation.

## Discussion

Adipocutaneous flaps and the McFarlane flap are widely used for investigating cutaneous microcirculation, angiogenesis, and pharmacological interventions. This is primarily due to their reproducibility and predictable necrosis patterns. However, the metabolic and vascular complexity of clinical reconstructions means that these random-pattern models have limited translational value. Musculocutaneous flaps offer a more representative platform in this regard, as the muscle component provides greater metabolic demand and closer similarity to human myocutaneous flaps, as well as higher ischemic tolerance. Therefore, while cutaneous models remain valuable for studying superficial perfusion and necrosis, musculocutaneous flaps are particularly important for addressing ischemia–reperfusion injury, systemic responses, and clinically relevant reconstructive (arterial and venous anastomosis) scenarios^7^^,^ 34 ^–^ 36.

The cutaneous maximus muscle in rats is a thin sheet-like muscle covering the lateral thoracic and abdominal wall from the shoulder to the root of the tail. The muscle is thickest at its dorsal origin near the axilla and gradually widens and thins into an aponeurosis as it approaches the *linea alba*. Li et al.^34^ also used this muscle for free flap transplantation. In contrast to their study, we preserved the pectoral muscles during the operation, and the circulation of the limbs was completely intact.

We have found that the flap pedicle blood flow is lowered in the musculocutaneous flaps. The lower arterial blood flow rate could originate from the new anatomical position of the flap-supplying branch of the lateral thoracic artery and vein. In case of local flaps, it was close to the original anatomical position, but the preparation itself and the regeneration of the surrounding tissues might cause a moderate narrowing of the vessels. When the flaps were interpolated into a new position and the pedicle had a new subcutaneous position on the frontal chest wall, the blood flow could be altered.

In cases in which the flap was transplanted to the femoro-inguinal region by performing microvascular anastomoses, it meant a new anatomical geometry and environment, such as bending of the vessels and the presence of the vascular anastomoses themselves. Although blood flow rate in the flap pedicles was lower, it seemed to be sufficient to maintain tissue perfusion and did not cause microcirculatory deterioration. It is important to note that the flap’s blood supply is exclusively dependent on the pedicle’s vessels until the neovascularization occurs along the wound (suture line)^37^^–^ 39.

When the flap was harvested and the excisions were made, all the collaterals were closed. These might contribute to the new perfusion and microcirculatory pattern. The 14-day follow-up period may have influenced the observed reduction in blood flow, as this time point often coincides with the transition from early hyperemia and sprouting angiogenesis toward vascular pruning and maturation. In addition, the early decrease in perfusion can also be attributed to tissue atrophy, which in this case may have been further aggravated by the absence of nerve repair, leading to denervation-induced muscle wasting and reduced metabolic demand. Consequently, lower perfusion values at two weeks do not necessarily indicate impaired vascularization but rather reflect the combined effects of vessel remodeling and tissue atrophy. Extending the follow-up to later time points, such as 28–42 days, would likely provide a more comprehensive picture, as vascular networks tend to become more mature, efficient, and stable over time, potentially revealing enhanced outcomes in terms of both vessel quality and functional perfusion capacity^40^^–^ 42.

There are several advantages of this model. In case of skilled microsurgeons, the model can be easily prepared and standardized, and it does not cause functional problems for the animals, compared to other musculocutaneous flap types, such as latissimus dorsi or other muscle-based flaps^5^^,^ 6 ^,^ 9 ^,^ 11. The supplying artery (lateral thoracic artery) can be gently mobilized, and it is long enough to transpose or transfer the flap. As this kind of flap can be prepared bilaterally, it provides an opportunity to compare various flap operation types, as it has been presented. However, there are a few disadvantages of the techniques: the flap size has a limit, and the thickness also influences the management of tissue defects. It is also important to mention that the cutaneous maximus muscle does not exist in humans. Therefore, the results are limited to rat models.

## Conclusion

The musculocutaneous flap based on the cutaneous maximus muscle in the rat can be applied and standardized for flap pathophysiology research. The tissue perfusion and microcirculatory pattern were sufficient for flap survival and the normal wound healing process. It is important to mention that appropriate microsurgical skill is essential for these experimental operations, and to obtain satisfying results a sufficient and safe learning curve is indispensable.

## Data Availability

The data will be available upon request.
